# The Book World of Medicine and Science

**Published:** 1904-10-08

**Authors:** 


### The Book World of Medicine and Science.

The Differential Diagnosis of Syphilitic and Non-Syphilitic Affections of the Skin, including Tropical Diseases. By George Pernet, Assistant to the Skin Department, University College Hospital. (London: Adlard and Son. 1904. Pp. 219. 8vo. Price 6s. 6d. net.)

Syphilis or not syphilis, as Dr. Pernet says, is a question which constantly intrudes itself in every department of medical practice, and it may be conceded that it is in the diagnosis of lesions of the skin that the problem presents itself most frequently and is most often difficult of solution. A correct diagnosis in these cases is of the utmost importance in view of the complex medical, social, and even legal questions involved, and this is only to be attained by a painstaking and complete examination, care being taken to avoid the common error of attaching too great value to one or two points to the exclusion of negative evidence. In this connection the chapter on general diagnosis is worthy of careful perusal and has an interest outside the domain of dermatology. The author's caution as to the use of the "therapeutic test" should also be remembered in view of the fact that many non-syphilitic affections are more or less benefited by mercurial treatment. Dr. Pernet discusses in turn the characteristic lesions met with under primary, secondary, tertiary, and congenital syphilis and contrasts them with the non-specific affections which are liable to be confused with them. Great pains have been taken to insure completeness in this respect and the author includes such tropical diseases as leprosy, yaws, and Oriental boil, liable to be met with in patients who have resided abroad. The frequency with which lesions occurring in unusual situations may lead to errors in diagnosis is well illustrated by some excellent remarks on extra-genital chancres, especially those affecting the fingers, the specific character of which even when occurring in medical men is liable to escape recognition till the appearance of a secondary eruption reveals their true nature. Another source of confusion is mentioned by the author which has received little attention though there is reason to believe it is much more frequent than usually supposed—the accidental inoculation of vaccinia in unusual situations. In this case the difficulty in diagnosis is usually not great, provided the possibility of such an occurrence is borne in mind. A useful description is added of the effects of conditions, syphilitic or otherwise, on the hair and nails, and also of the character of the scars left by various destructive processes, which often are of much diagnostic value. While the effects of the *x*-rays on the nutrition of the nails are described the loss of hair which frequently follows their use has not been included among the other forms of baldness. The book has been well printed in good clear type on excellent paper and has the advantage of an efficient and exhaustive index which should greatly facilitate its use as a work of reference. That Dr. Pernet's work fills a gap in medical literature is undoubted, but its value will be more apparent in its practical application to clinical teaching than as an aid to the diagnosis of individual cases.

Wounds in War: The Mechanism of their Production and their Treatment. By Surgeon-General W. F. Stevenson, C.B., A.M.S. (London: Longmans, Green and Company. 1904. Second Edition. Pp. 511. Price 15s. net.)

The appearance of this edition is welcome, and especially so as the experiences of the late war have provided much material on the subject at a time when it was becoming manifest that under conditions of modern warfare some changes in the arrangements for treating the wounded were desirable. General Stevenson enters fully into the mechanics of projectiles, a correct understanding of which is, of course, essential, and then gives a full and very lucid account of the wounds which they produce, at varying ranges, on the human tissues and the corresponding appropriate methods of dealing with the injuries. In no other branch of surgery has the antiseptic principle effected a greater revolution of practice. General Stevenson reminds us that it was formerly the rule to treat all gunshot wounds of large joints, without exception, by primary amputation, while it is now the rule, with, perhaps, not more than 2 per cent. of exceptions, never to perform primary amputation for these injuries. And, in the few exceptions, neither the degree of injury to bones nor the fact of a joint being opened are to be accepted as the guides to other than conservative measures; the decision to amputate will rest chiefly on the damage done to the main nerves and vessels of the limb. The author very rightly lays great stress on the necessity for massage and early movements in all injuries in which a joint is involved. A new chapter has been added on the use of *x*-rays in war hospitals, and is in great part based on actual experience in South Africa. Not the least instructive pages of the volume are those which are devoted to certain general considerations, such as Bearer Companies in the Field and the Geneva Convention. Throughout the entire treatise there is but little opportunity for fault-finding, and, indeed, it possesses the uncommon virtue that theoretical considerations are everywhere entirely subordinated to observed facts.

The British Journal of Inebriety. Vol. II., No. 2. (London: Baillière, Tindall, and Cox.)

The current issue of this journal contains a number of interesting articles. Dr. Harry Campbell contributes a series of notes on the drinks of pre-agricultural man, in which he suggests that the pre-agriculturists failed to hit upon the plan of brewing alcoholic drinks, probably because of the non-possession of convenient vessels for holding liquids, especially such as could withstand fire. From the same pen is an able and closely-reasoned argument in support of Dr. Archdall Reid's position on inheritance and environment in relation to the alcohol question. This article is an example of the manner in which the alcohol question should be treated, in a journal professing to be devoted to scientific study.

In view of the position which most of the writers adopt in reference to the failure of moral suasion as a cure for the excessive use of alcohol, it is somewhat surprising to find how confidently they advance the same agency in restraint of the sexual instinct. Inebriates, we are told in one article, should be made to "understand that they are under a great moral obligation not to help to people the world with unfit beings;" on another page we read of the risks of pregnancy in the reclaimed female inebriate and are advised that this condition should not "be encouraged." An article having wide interest is that by Dr. Wm. C. Sullivan on "The Criminal Responsibility of the Alcoholic," in which chronic alcoholic insanity, delirium tremens, and the dream-consciousness of morbid drunkenness, are discussed in their several relations to homicidal offences. Dr. T. D. Crothers also contributes a suggestive paper on "Alcoholism and Inebriety." Altogether the present issue of the Journal well represents the activity and energy of the Society of which it is the organ. That Society has an opportunity for a large scientific and social influence. But to attain this its work must be conducted on rigidly scientific lines.

